# News and Notes

**Published:** 1904-05

**Authors:** 


### *NEWS AND NOTES.*

It will be of interest to all physicians in the State to learn that a professional nurses' register has been established at Palmer's Viaduct Pharmacy, 11 Peachtree street, Atlanta. Only graduates and competent names are on this register, and one can be obtained by either writing, telegraphing or telephoning them. Phone 64.

---

A Resident Physician is wanted for the Tabernacle Infirmary. One having some acquaintance with bacteriology and pathology preferred. Address or apply to Dr. R. R. Kime, English-American Building, Atlanta, Ga.

---

Two distinguished physiologists, Prof. Luigi Luciani (of the University of Rome) and Prof. Angelo Mosso (of the University of Turin), have been named Senators of the Kingdom of Italy.

---

Prof. Guido Bacelli of the University of Rome, who has for several years past held a portfolio in the Italian government, first as Minister of Public Instruction and later as Minister of Agriculture, has lately resumed active work as a clinical teacher.

---

The Johns Hopkins University Hospital suffered severely in the recent terrible fire at Baltimore by the destruction of a house property from which a considerable portion of its revenues were drawn. Mr. J. D. Rockefeller has given $100,000 to make good the loss caused to the hospital.

---

The trained Chimpanzee "Consul," valued at $125,000, succumbed recently to an acute affection at Berlin. He put up at good hotels and always had a room to himself, with engagements booked for two years in advance at a monthly salary of $4,000 and his life was insured for $100,000.

---

THE Seventh International Congress of Hydrology and Climatology will be held at Venice in September, 1905. The Italian Minister of Public Instruction, Prof. V. E. Orlando, is president of the honorary committee, and Prof. De Giovanni, of Padua, is president of the organizing committee.

---

THE fifth International Congress of Dermatology will be held at Berlin this year from September 12 to 17. The following questions are proposed for discussion: (1) Skin affections in anomalies of metabolism; (2) Epitheliomata and their treatment; (3) Syphilitic diseases of the circulatory system; (4) *a*, The state of the question of the spread and prevention of leprosy since the date of the first leprosy conference held in 1897; *b*, The present state of knowledge concerning anesthetic leprosy.

---

THE whole region of Northeastern Siberia, from the Yakutsk territory down to Mongolia, is said to be suffering from a visitation of smallpox. Medical men are few and far between in the affected district. In many cases the district surgeon has to visit settlements which are distant one hundred miles and even more from his house. Mongolians gladly allow themselves to be vaccinated, but there is a difficulty in procuring vaccine, and much of what is obtained is said to be rendered useless by the cold to which it is exposed in transit.

---

IN these days original Japanese contributions to medical science are taken as a matter of course, yet it is worthy of note, says a contemporary, that it was a recognition of the superiority of Western medicine that first led the Japanese to study and adopt European life, language and methods. Before the year 1867 the only European language known to the Japanese was Dutch, which was studied by the interpreters as a medium for acquaintance with Western medical science. Possibly the choice of Dutch may ultimately be traced to the influence of Boerhaave, the famous physician of Leyden, from whom, as it happens, Peter the Great took lessons in 1715.

---

THE *Journal of the American Medical Association* states that the Russian Red Cross Society was founded in 1860 at Moscow, and now has more than 600 organized committees, forty-two hospitals, two training schools for nurses, eight soldiers' homes, and two sanatoria for children. There are more than 4,000 women nurses and a battalion of men nurses. Our Russian exchanges have contained of late many notices of the way in which relief work for the war has been systematically proceeding. The Red Cross supplies at Charbin now include nearly $40,000 worth of materials for dressings; they have medical supplies for 30,000 beds. Besides this, twenty-six field hospitals have been set up, with 5,050 beds. To each 500 beds there are five physicians, one administrator, fifteen women nurses and thirty other attendants. Six of these have already been installed at Charbin. The society is being constantly and lavishly supplied with gifts of linen, preserves, wines, and other delicacies, with contributions of money. Holland is preparing a field hospital, and another is being equipped by Russian residents at Berlin, which will soon start for the seat of war.

---

AN exchange says: "Professor Royce, recently returned from Egypt, gave an interesting account of the success which has attended the efforts to extirpate the mosquitoes and malaria from Ismailia. He said that when Major Ross visited Ismailia in September, 1902, there were 2,000 cases of malaria annually in a population of 9,000 people, of whom 2,000 were Europeans. The authorities at Ismailia loyally carried out his suggestions as to filling up marsh land close to the town and cleaning out small irrigating channels and stagnant waters. That involved an expense of $22,000, and at the same time they organized a drain brigade and a petroleum brigade, and now people could sleep in any of the houses in the European quarter without mosquito nets. Malaria cases had been reduced from 2,000 a year to 200. As a matter of fact, there were no fresh cases of malarial infection in Ismailia; there had been no deaths among Europeans during 1903, and only four among natives, against thirty deaths the year before. The improvement was so wonderful that Prince D'Arenberg, President of the Suez Canal Company, hopes soon to see Ismailia the sanatorium and watering-place for Cairo. Major Ross, who was present, remarked that the success of the anti-malarial campaign at Ismailia had taught two things—that it was possible to rid a large town entirely of mosquitoes, and that it was equally possible to eradicate malaria. He had been asked to draw up a report as to malaria cases in India, which were responsible for 300,000 admissions to hospitals from the troops and jail prisoners. With the Ismailia figures before him, he felt confident that these high figures would soon be reduced by at least one-third."

